# Electro-mechanical behaviour of mortars reinforced with alternative electrically conductive inclusions

**DOI:** 10.1617/s11527-025-02590-4

**Published:** 2025-02-04

**Authors:** Niki Trochoutsou, Danny Smyl, Giacomo Torelli

**Affiliations:** 1 Department of Civil and Structural Engineering, The University of Sheffield, Sheffield, UK; 2 School of Civil and Environmental Engineering, Georgia Institute of Technology, Atlanta, GA USA

**Keywords:** Self-sensing, Composites, Smart, Carbon fibres, Recycled carbon fibres, Graphite powder

## Abstract

The incorporation of electrically conductive inclusions in structural materials can impart self-sensing functionalities, making them ideal for structural health monitoring applications. However, the use of more sustainable alternatives and their effect on key engineering properties remain largely unexplored, while the adoption of different testing protocols for the characterisation of electrical/self-sensing properties can lead to different results, thus questioning their reliability, even for existing smart composites. This paper investigates systematically the effect of recycled carbon fibres and graphite powder on the mechanical, electrical, transport properties and piezoresistive performance of cementitious mortars. Virgin carbon fibres, at dosages equivalent to those of recycled fibres, were also examined to establish a performance benchmark. Fibre content ranged from 0.05% to 1% vol., while graphite powder was added as sand replacement at contents varying from 0.3% to 3% vol. The effect of existing testing protocols and electrode layout on the piezoresistive performance was also examined, and the associated limitations and challenges are discussed in detail. The results demonstrate the potential of recycled carbon fibres as a cost-effective alternative in smart applications, without compromising electrical and piezoresistive performance. The use of 0.25%vol. of recycled or virgin carbon fibres was found to provide the desirable synergy between structural performance, cost and self-sensing properties, yielding a 50–60% increase in flexural strength, and good piezoresistivity with a gauge factor of 90–110. In contrast, the use of graphite powder resulted in composites with poor self-sensing ability even at the highest content examined (3%vol.), also accompanied by a reduction in compressive strength up to 33%.

## Introduction

Incorporating electrically conductive constituents in the form of particles (e.g. carbon black, metal/graphite powder) [1, 2] or fibres (e.g. micro- and nano-fibres including carbon/steel fibres, carbon nanotubes) [1, 3–7] into cementitious composites offers a promising approach for developing self-sensing materials with tailored electrical properties. By leveraging the piezoresistive effect, where applied mechanical strain can be transduced into a measurable variation of electrical resistance, structural elements can be transformed into distributed sensors, thus providing significant potential for structural health monitoring applications [8, 9].

Among the particle-based inclusions, graphite powder (GP) has more recently received considerable attention due to its favourable cost-performance ratio and wide availability [10], along with its ability to impart higher conductivity in cementitious composites when compared to other particle-based inclusions [11]. The majority of studies have focused on the effect of increasing contents on the basic electrical and mechanical properties, identifying the percolation threshold [10–14], i.e. the minimum conductive inclusion content required for the formation of a continuous, or percolating, conductive network [15], in the range of 1–15%wt. binder. However, the lack of a systematic investigation on the combined electrical, mechanical, and transport properties of mixtures containing GP, along with its inconsistent use as cement or sand replacement and lack of well documented mix designs, further complicates the understanding of the underlying microstructural and electrical mechanisms and hinders the identification of the optimal content for a desirable functional response. Only one study [13] assessed the self-sensing properties of lime mortars doped with GP and concluded that high piezoresistivity can be achieved with 10%wt. binder.

Carbon fibres (CF) represent the most comprehensively studied fibre-type inclusion due to their high resistance to corrosion, thermal stability, and lower cost when compared to carbon nanotubes [16], while also improving the mechanical properties of the resulting composites by providing a crack bridging effect. However, the complex interdependency between piezoresistive properties and composition of the host material, coupled with the lack of standardized testing methodologies, resulted in a wide range of percolation thresholds (0.2% to 1% vol.) [15, 17, 18].

While the demand for CF in various sectors is increasing at a rapid pace, it is estimated that approximately 30% of the global CF production ends up as waste [19], evidently posing significant environmental challenges [20]. The use of recycled carbon fibres (RCF) can offer a very promising alternative. Depending on the recycling process, RCF can be approximately 30–40% less expensive than virgin CF, while retaining up to 90% of their original strength and stiffness [21]. Segura et al. [21] and Faneca et al. [22] provided encouraging evidence that RCF contents between 0.2 and 0.8% vol. may result in low resistivity composites (3–0.6 Ωm), while Belli et al. [7, 23] reported similar percolation thresholds for mixes containing virgin CF and RCF (approximately 0.1–0.2%), which however did not result in improved piezoresistivity. Despite their great potential, research on RCF cementitious composites is extremely limited, and there is still lack of fundamental understanding on how to design these composites to simultaneously satisfy mechanical, electrical, and durability requirements while guaranteeing appropriate piezoresistive properties. In addition, the considerable variations in properties and surface characteristics of RCF due to their diverse origin and recycling processes [24] hinders reliable comparisons of existing experimental data.

This study provides a comprehensive examination of the effects of RCF and GP on the combined mechanical, electrical, durability and piezoresistive response of cementitious mortar mixes. The use of virgin CF at dosages equivalent to those of RCF is also examined to establish a performance benchmark. By systematically varying the content of conductive inclusions, this research aims to elucidate the underlying microstructural mechanisms governing the composite performance, and explores the potential of these engineered composites as multifunctional materials. A comparative analysis between the two- and four-probe method by varying the electrode layout was also undertaken and the limitations and challenges related to the implementation of established self-sensing characterisation methodologies are commented upon. The present paper directly contributes to the development of alternative, more sustainable and cost-effective functional cementitious composites for smart applications.

## Materials and methods

### Materials and specimen preparation

A total of 17 different mortar mixes were tested in this study, including one plain reference mix (i.e. without electrically conductive inclusions). Manufactured polyacrylonitrile-based CF and RCF with glycerine sizing and similar characteristics (Table 1) were incorporated into the mixes at volume fractions ranging from 0.05% to 1%. Due to its inert nature, GP was added as sand replacement at volume fractions ranging from 0.3 to 3% to minimize impact on mechanical performance, yet enabling direct comparison with the majority of existing studies [e.g. 11, 12]. A high fineness expanded GP was selected to minimize the quantity of inclusions required to achieve the percolation threshold without compromising mechanical performance [10].

**Table 1:** Characteristics of CF, RCF and GP, as provided by the suppliers

Property	CF	RCF	GP
Diameter (μm)	7	7	20^*^
Length (mm)	6	6	–
Specific Gravity (g/cm^3^)	1.8	1.7–2.0	2.2
Tensile Strength (MPa)	4000	3500	–
Young’s Modulus (GPa)	240	230	–
Carbon Content (%)	> 99	> 94	> 95
Electrical Resistivity (μΩm)	15	15	1

*Value refers to the median diameter, d_50_

All following discussion is based on the performance of fibre- and particle-based inclusions separately, due to their different aspect ratio and the different impact on mechanical, microstructural and electrical performance.

**Fig. 1:**
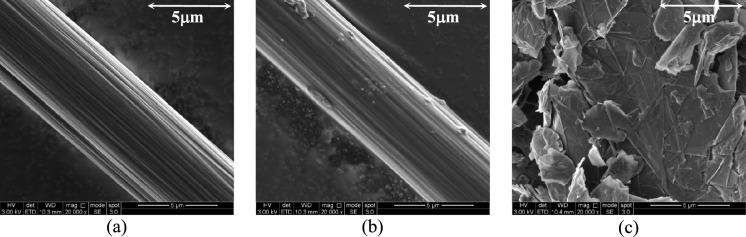
SEM images of exemplary inclusion samples: **a** CF; **b** RCF; **c** GP

The morphology of the tested inclusions and resulting composites was examined using Scanning Electron Microscopy (SEM) with a FEI Inspect F50. While CFs exhibit a smooth surface (Fig. 1a), RCFs are characterized by marked roughness and surface defects (Fig. 1b), possibly due to resin residues from the recycling or milling process [25]. GP has a flaky crystalline structure and consists of thin plate-like particles, typically folded and wrinkled (Fig. 1c).

All mortar mixes were produced using CEM I 52.5 N and fine aggregates with a maximum size of 2 mm. A water/cement ratio of 0.5 by weight and a fines/cement ratio of 0.75 by volume were used across all mixes to ensure the same composition of the mortar matrix, and attain electrical continuity of the cement (cement percolation) in the presence of sand [15]. As the mixes with higher fibre and GP quantity showed higher water demand, a superplasticizer was used to achieve similar workability (with target flow values in the range of 100–150 mm, measured in accordance with EN 1015-3 [26]) without altering the target w/c ratio, in line with previous studies [27, 28]. To ensure a reliable comparison between mixes including CF and RCF, the amount of superplasticiser used for each of the fibre contents examined was kept constant across the two fibre types, while the mix composition was identical.

**Fig. 2:**
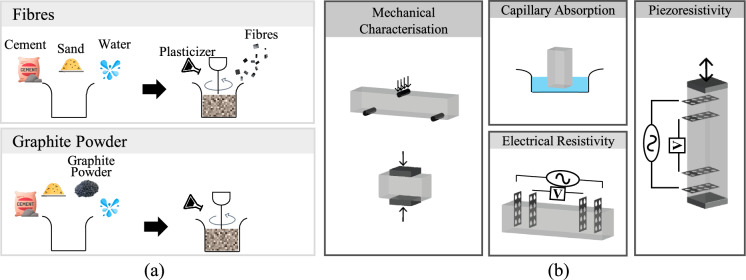
**a** Mortar mixing method; **b** summary of experimental programme

Two different mixing processes were followed, as illustrated in Fig. 2a. Fibres were incorporated into the mix by implementing a wet mixing method to ensure better fibre dispersion and piezoresistive response [22, 29]. By contrast, GP was incorporated into the mix together with all the dry materials, as this was proven to ensure uniform dispersion of GP particles with ordinary mechanical means [13]. After casting, all specimens were cured in a mist room (20°C ± 2°C, RH = 99%) for a period of 28 days. A summary of the experimental programme is illustrated in Fig. 2b with further details given in the following sections.

### Characterisation tests

#### Mechanical tests

The flexural, ${f}_{ct,fl}$, and compressive strength, ${f}_{c}$, of the mortars were experimentally determined at 7 and 28 days through three-point bending tests on 160 × 40 × 40 mm prisms and compression tests on the resulting halves, in accordance with EN 1015-11 [30]. The post-cracking flexural response of all mixes was assessed at 28 days through three-point bending tests on notched prisms of the same size, following EN 14651 [31]. The crack mouth opening displacement, *CMOD*, was measured by means of an extensometer spanning across the two sides of the notch. The flexural strength and the fracture toughness ${K}_{IC}$ were calculated according to the RILEM TC265-TDK recommendation [32].

#### Capillary water absorption tests

The water absorption under capillary action was assessed in accordance with EN 1015-18 [33]. After the end of the 28d curing period, six half-prisms for each mix were dried at 60ºC until weight stabilisation, and subsequently immersed in water. Weight measurements were recorded after 10 and 90 min of immersion, and the capillary water absorption coefficient, *C*, was determined.

#### Electrical resistance measurements

**Fig. 3:**
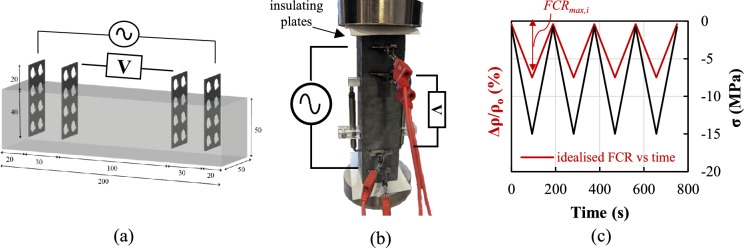
**a** 3D scheme of the electrode layout (dimensions in mm); **b** experimental setup for piezoresistivity testing; **c** loading protocol

The electrical resistivity of the composites was measured at 7 and 28 days using the four-probe method [34] on 200 × 50 × 50 mm saturated specimens equipped with perforated stainless steel electrode plates. In the absence of standardized guidelines, the electrode layout adopted in this study (Fig. 3a) was informed by other four-probe setups described in the literature. Specifically, since relatively small distances between electrodes may lead to inconsistent resistivity measurements [35, 36], a relatively large distance between current and voltage pole (> 7.5 mm) was used. The distance between inner electrodes was always kept larger than 60 mm, while the distance between adjacent electrodes was kept greater than four times the fibre length (i.e. > 24 mm) to ensure adequate fibre distribution [37].

A low amplitude, high frequency (1 mA, 100 kHz) alternating current was adopted to limit the contact resistance effect at the electrode/matrix interface [35], minimize polarisation effects [37], and avoid Joule heating. The derived ohmic resistance, *R*, was converted to electrical resistivity, *ρ*, following Eq. (1):

1 $$\rho =R*\frac{A}{L}$$

where, *A* is the cross-sectional area of the specimen and *L* is the distance between the inner electrodes.

#### Piezoresistivity tests

The piezoresistive behaviour of the composites was investigated by continuously monitoring the variation in electrical resistivity of specimens (200 × 50 × 50 mm) subjected to cyclic compression loading. The load was applied at a rate of 200 N/s and the electrical resistivity was measured using both the two- and four-probe methods to assess the reliability of the obtained data. For the former, the inner voltage poles were used for both current injection and voltage measurement, while for the latter, the same setup and methodology described in Sect. 2.2.3 were employed (Fig. 3b). Each test comprised four loading–unloading cycles up to a maximum load inducing a stress of 15 MPa, equivalent to approximately 30% of the specimens’ average strength, starting from a 2 kN preload (Fig. 3c). Two LVDTs were placed on opposite sides of each specimen to measure uniaxial deformation and derive average strain values.

The corresponding fractional change of electrical resistivity, *FCR*, was calculated as *Δρ/ρ*_*ο*_, where *Δρ* is the difference between the resistivity measured throughout the test and the initial resistivity *ρ*_*ο*_. Figure 3c illustrates the qualitative evolution of FCR in the ideal scenario where the resistivity changes linearly with compressive strain. The piezoresistive performance is then assessed through the analysis of the following four metrics: i) *FCR*_*max*_, taken as the largest change of electrical resistivity during cyclic compression; ii) Gauge Factor, *GF*, computed as the ratio of *FCR*_*max*_ to the corresponding applied strain (i.e. *FCR*_*max*_*/ε*); iii) repeatability, *L*, calculated as (*FCR*_*max*_*-ΔFCR*_*max,i*_*)/FCR*_*max*_, where *ΔFCR*_*max,i*_ is the maximum deviation of *FCR* values under the same compressive strain in all loading cycles, and iv) linearity, expressed as the R^2^ error, calculated from the best fitted line against the FCR versus *ε* data.

## Results and discussion

### Mechanical properties

Table 2 summarises the average values along with their coefficient of variation, *CoV*, (given in parentheses). Specimens exhibiting properties within one standard deviation of the mean were considered to be statistically significant, else they were treated as outliers.

**Table 2:** Mechanical, absorption and electrical properties of mortars. Values at 28 days, unless otherwise stated, with CoV in parentheses

Specimen ID	Content (%vol.)	f_ct,fl_ (MPa)		f_c_ (MPa)		CMOD (mm)	E (GPa)	K_IC_ (MPa√m)	C (kg/(m^2^√min))	ρ ($\Omega$cm)	
		7d	28d	7d	28d					**7d**	**28d**
M-REF	-	5.7 (16%)	5.6 (5%)	46.2 (3%)	71.4 (3%)	0.016 (1%)	27.0 (17%)	1.43	0.309 (20%)	1918.7 (6%)	2308.0 (4%)
M-CF-	0.05	6.8 (2%)	6.3 (3%)	36.7 (2%)	54.8 (2%)	0.019 (20%)	23.0 (3%)	1.60	0.504 (10%)	116.1 (8%)	151.4 (10%)
	0.1	9.9 (1%)	6.2 (0%)	43.7 (1%)	60.8 (4%)	0.042 (2%)	27.1 (10%)	1.58	0.595 (8%)	48.2 (4%)	55.0 (4%)
	0.25	10.1 (2%)	8.3 (3%)	43.1 (3%)	62.5 (2%)	0.057 (13%)	26.0 (3%)	2.19	0.588 (7%)	19.2 (11%)	23.2 (9%)
	0.5	10.0 (0%)	12.2 (1%)	41.9 (4%)	60.8 (5%)	0.064 (0%)	20.9 (7%)	3.08	0.509 (5%)	7.0 (6%)	8.6 (7%)
	0.75	13.7 (2%)	13.8 (14%)	44.3 (4%)	62.4 (2%)	0.111 (1%)	22.4 (7%)	3.49	0.430 (1%)	6.8 (7%)	8.3 (8%)
	1	14.5 (17%)	17.6 (0%)	41.0 (2%)	63.1 (1%)	0.086 (7%)	25.4 (0%)	4.44	0.561 (4%)	4.3 (20%)	4.8 (20%)
M-RCF-	0.05	6.6 (6%)	5.4 (3%)	42.4 (1%)	56.5 (5%)	0.016 (5%)	19.5 (3%)	1.36	0.395 (4%)	645.5 (7%)	848.2 (6%)
	0.1	9.1 (2%)	4.6 (5%)	40.8 (4%)	55.5 (4%)	0.067 (45%)	15.5 (5%)	1.14	0.654 (5%)	79.2 (3%)	95.2 (5%)
	0.25	8.3 (2%)	9.3 (1%)	42.4 (5%)	69.1 (3%)	0.044 (1%)	24.3 (0%)	2.36	0.595 (2%)	23.5 (5%)	38.0 (2%)
	0.5	13.4 (0%)	13.7 (5%)	43.6 (2%)	68.1 (3%)	0.058 (17%)	23.2 (5%)	3.46	0.457 (4%)	6.7 (5%)	8.3 (3%)
	0.75	14.7 (2%)	14.2 (5%)	40.6 (1%)	62.4 (2%)	0.089 (5%)	24.7 (4%)	3.60	0.440 (5%)	4.6 (3%)	4.7 (7%)
	1	16.1 (5%)	17.5 (2%)	43.8 (1%)	70.7 (1%)	0.131 (10%)	26.7 (5%)	4.42	0.332 (3%)	4.7 (8%)	5.2 (9%)
M-GP-	0.3	7.6 (4%)	5.6 (3%)	41.7 (6%)	59.9 (2%)	0.011 (2%)	22.9 (2%)	1.43	0.439 (6%)	1805.0 (3%)	2289.7 (1%)
	0.7	7.7 (4%)	5.3 (2%)	40.1 (4%)	57.7 (5%)	0.014 (0%)	21.1 (9%)	1.35	0.294 (11%)	1442.1 (3%)	1818.8 (1%)
	1.5	7.1 (1%)	4.3 (8%)	37.3 (1%)	53.5 (4%)	0.016 (6%)	20.6 (15%)	1.07	0.186 (10%)	770.1 (3%)	1047.2 (1%)
	3.0	7.7 (1%)	7.7 (5%)	32.6 (1%)	48.0 (3%)	0.020 (6%)	17.6 (7%)	1.94	0.156 (13%)	245.3 (5%)	329.2 (5%)

#### Compressive strength

**Fig. 4:**
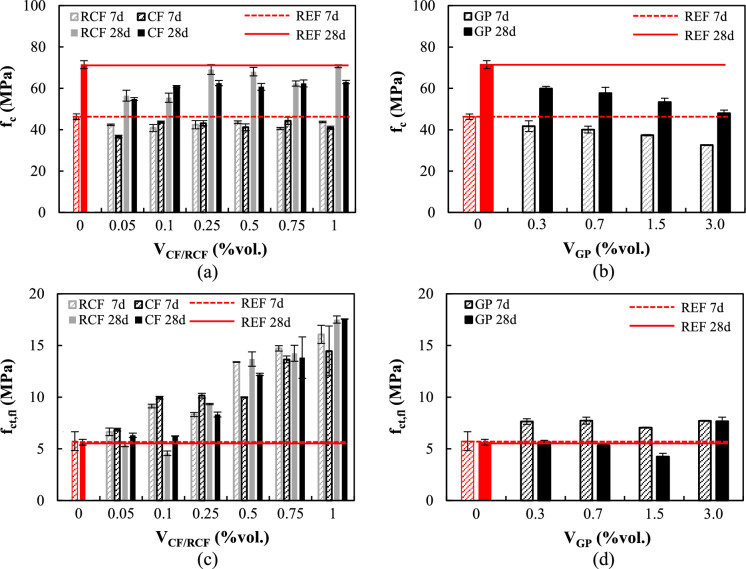
Compressive and flexural strength of mortars versus inclusion content: **a**, **c** CF/RCF, **b**, **d** GP. Flexural strength values were obtained at 7 and 28 days on un-notched and notched specimens, respectively

**Fig. 5:**
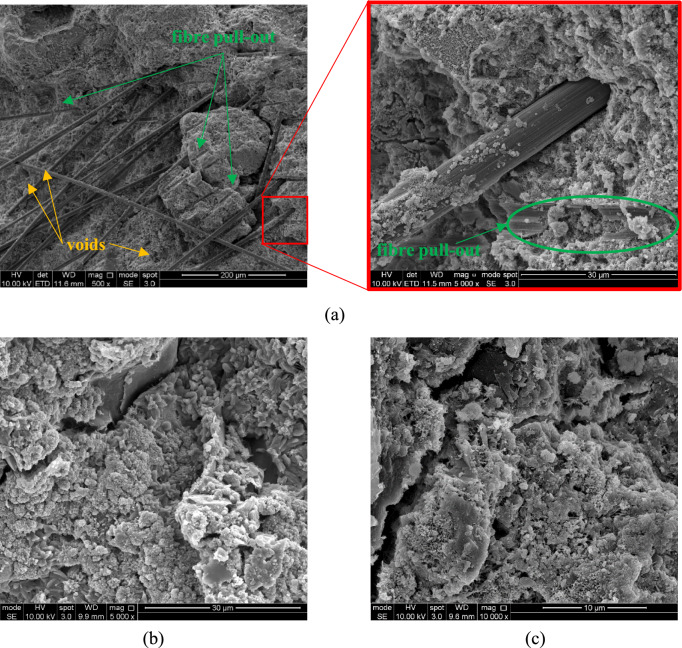
SEM images of mortar with **a** 0.75% vol. RCF; **b** 1.5% vol. GP; **c** 3% vol. GP

The effect of fibres and GP on the 7- and 28-day compressive strength, *f*_*c*_, of the resulting mortars is shown in Fig. 4a,b, along with the average properties of the reference mix (M-REF). The strength development of the mixes containing the examined inclusions was observed to be generally close to that of M-REF. However, mixes with inclusions exhibited a slight reduction in compressive strength compared to that of M-REF. Specimens doped with CF and RCF exhibited a 28-day strength reduction up to 20%, with the largest reduction being observed for the low fibre content mixes (up to 0.1% vol.). Interestingly, the use of higher RCF contents did not seem to affect compressive strength, with the recorded 28-day average values being only 1–5% lower than that of the M-REF (Fig. 4a). The reduction in compressive strength could be due to fibre agglomerations and reduced workability at increasing fibre contents, which despite the use of superplasticiser, resulted in lower compaction and the formation of air voids (Fig. 5). As will be further discussed in Sect. 3.2, mortars doped with lower contents of CF and RCF presented a higher volume of absorbed water than the M-REF, indicating a more porous microstructure.

Achieving uniform fibre dispersion is undoubtedly a challenge. It should be reminded that a wet-mixing method was employed in this study, as it is cost-effective when compared against pre-mixing or co-mixing methods and avoids the use of admixtures that could potentially affect the matrix development [24] or special equipment (e.g. ultrasonication), yet ensuring adequate dispersion [22, 29]. However, optimisation of the fibre dispersion should be further explored, either at a preparation stage, with the use of appropriate dispersant or surface treatment, or at a mixing stage, with the implementation of pre-mixing or intermediate wet-mixing, dry–wet-mixing, and wet-mixing steps [38]. In parallel, the investigation of the compatibility of novel dispersants with recycled carbon fibres of diverse origin/recycling processes, as well as of the sizing applied during manufacture on the fibre dispersion, is imperative for the development of composites with superior mechanical properties and a stable, well-connected conductive network.

The mortars doped with GP exhibited an almost linear decrease in compressive strength at increasing GP contents (Fig. 4b), down to 33% of that of M-REF (at 3% vol.). As GP acts as an inert filler and does not contribute to cement hydration [10], the reduction in strength, also seen in previous studies (e.g. [11]), can be attributed to a combination of three main mechanisms: (a) the lamellar structure (Fig. 1c) and intrinsic lubricating function of GP reduce friction resistance between the graphite particles, hindering stress transfer within the composite [39]; (b) graphite particles tend to agglomerate and encapsulate cement particles, thus inhibiting cement hydration [40]; (c) the lower hydrophilicity of GP compared to cement weakens the interfacial bond [41]. Figure 5b,c show the SEM micrographs of mortars doped with GP, where individual flakes are hardly visible due to the formation of hydration products.

Detailed analyses on the electrokinetic mechanisms of the GP powder, through zeta potential characterisation techniques, should also be performed to gain insights into the dispersion quality and propensity to agglomeration and aid the selection of the optimal GP powder and content according to the application.

#### Flexural strength

The effect of the inclusions on the 7- and 28-day flexural strength, *f*_*ct,fl*_, is summarised in Fig. 4c, d. It should be noted that, while the 7d strength was determined on un-notched prisms, the 28d strength was determined on notched prisms and any differences between the two test groups can be associated mainly with the different test setup rather than the effect of the inclusions on strength development [42, 43].

An almost linear enhancement in flexural strength was recorded with the addition of CF/RCF at fibre contents > 0.25%, both at 7 and 28 days. This indicates that both types of fibres can effectively control crack initiation and development, owing to their good bond and bridging action. At low fibre contents (≤ 0.1%vol.), a reduction in strength by up to 20% was recorded for RCF-doped specimens. This could be attributed to the low fibre content, which was insufficient to provide any effective strengthening of the matrix, and the possibly high amount of air voids entrapped within the mix, as also confirmed by the higher water absorption (Fig. 7). Hence, the minimum CF/RCF fibre content required to enhance flexural strength can be taken as 0.25%vol. In all cases, failure of the specimens was a result of fibre pull-out, as evident from the analysis of the fractured surface (Fig. 5a). This promoted the progressive engagement of the fibres intersecting the fractured surface and resulted in an increase in flexural strength with increasing fibre content.

Although a decrease in compressive strength was observed at increasing GP contents (Fig. 4d), the incorporation of a low content of GP (≤ 0.7%vol.) did not lead to a reduction in the 28d flexural strength, while a more evident variation was recorded for a content of 1.5%vol. (−25%, 4.3 MPa), and 3% vol. (+ 36%, 7.7 MPa). The increased flexural strength of composites doped with 3%vol. GP could be attributed to the increase in packing density, as also evidenced by their lower porosity (see also Fig. 7).

#### Flexural response and fracture properties

**Fig. 6:**
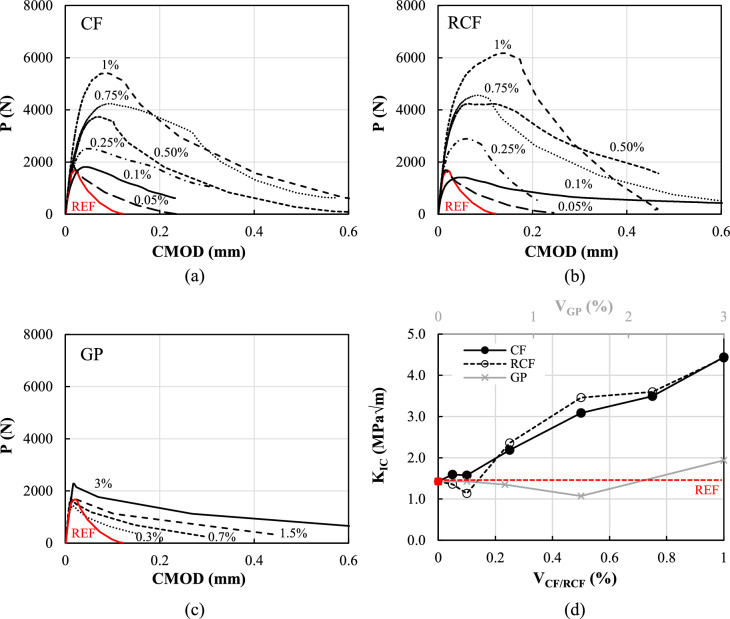
**a**–**c** Typical load-CMOD curves for different contents of CF, RCF and GP; **d** fracture toughness versus inclusion type and content

The complete load-CMOD response of representative specimens made with mixes comprising the examined inclusions is shown in Fig. 6a–c, along with the behaviour exhibited by a representative specimen made with the reference mortar (red curves). The average values of CMOD at maximum load, elastic modulus in flexure and unstable fracture toughness, are summarised in Table 2, along with their coefficient of variation, *CoV* (given in parentheses).

Specimens including fibres (CF/RCF) showed a linear behaviour up to cracking, followed by a non-linear behaviour up to the maximum load, and a strain-softening branch (Fig. 6a,b). As expected, the initial stiffness of the reinforced composites was comparable to that of the reference regardless of the fibre content and the type of fibre (CF or RCF). The post-peak softening branch is the result of fibre pull-out and the potential fibre shearing upon crack development [44]. Higher CMOD values before failure were obtained with increasing fibre content, due to the favourable effect of the random fibre distribution (Fig. 5a) and the ability of the fibres to control crack development.

In contrast, the flexural performance of specimens with GP was characterised by a more brittle post-peak behaviour, though still exhibiting relatively higher post-peak residual strength and CMOD values compared to M-REF, indicating that GP was able to provide a degree of resistance to crack propagation, albeit limited when compared to fibre-based inclusions due to its smaller aspect ratio.

*K*_*IC*_ was also analysed to quantify the ability of the material to control crack growth as a result of the bridging effect of the fibres and the interlock of the microstructure (Fig. 6d). It can be clearly seen that the values of *K*_*IC*_ notably increased with increasing CF/RCF quantity, especially beyond 0.25% vol. For the highest fibre content examined (1%vol.), *K*_*IC*_ values approximately three times greater than that of the M-REF were obtained. As discussed above, the fracture toughness of GP-doped composites was not affected critically by increasing GP content when compared to M-REF, confirming the limited ability of GP to effectively prevent crack propagation.

### Capillary water absorption

**Fig. 7:**
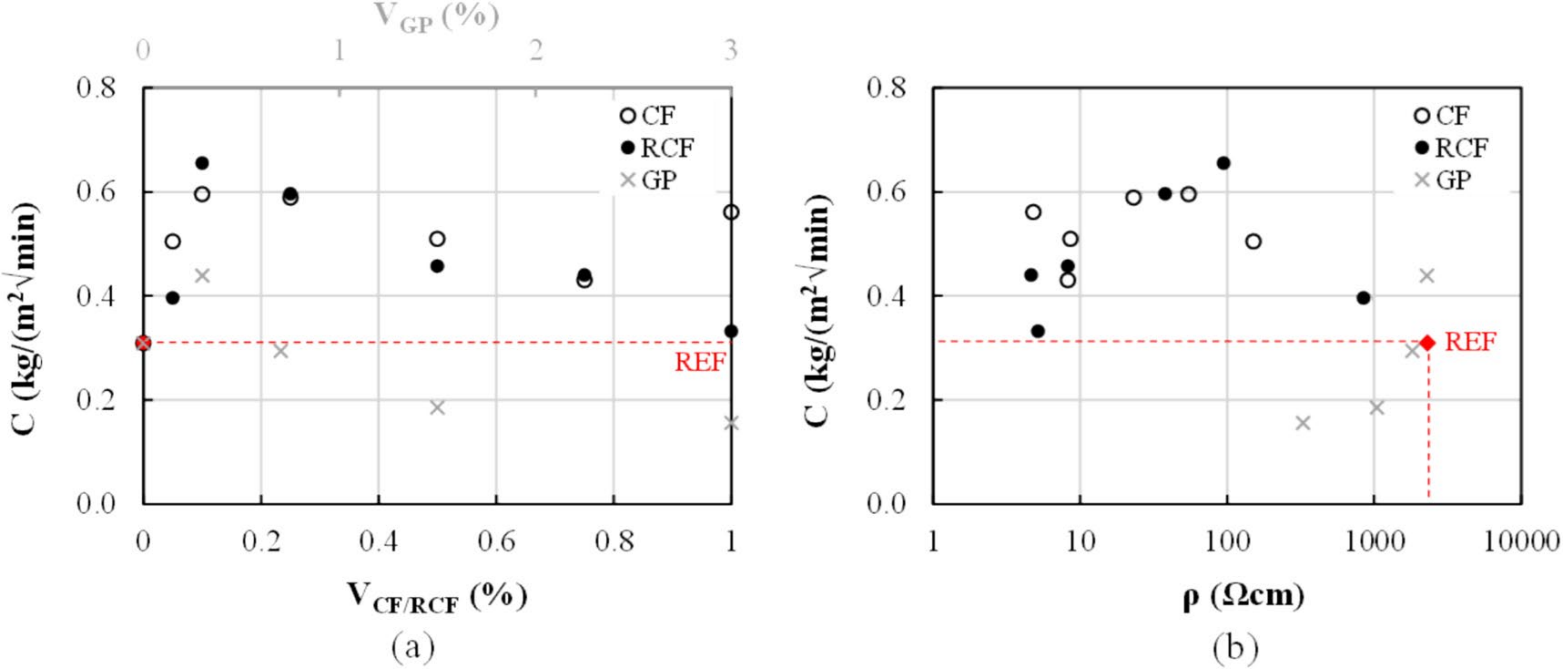
**a** Effect of inclusions on capillary water absorption; **b** Capillary water absorption and associated 28d electrical resistivity

Overall, the mortars doped with CF/RCF fibres exhibited a higher water absorption capacity than the M-REF (Fig. 7a), which indicates a reduction in their potential durability. The development of a more porous microstructure can be attributed to the presence of a higher amount of air entrapped in the mix as a result of fibre agglomeration, which also affected the compressive strength (Fig. 4a). However, reduction in capillary water absorption can be observed at increasing fibre contents, probably as a result of the combined action of: a) the fibres, in decreasing internal microcracking (due to shrinkage [45]) and in disrupting the continuity of the capillary pores; b) the use of a higher dosage of superplasticiser, which promoted a higher degree of compaction and a reduction in the size of the capillary pores, while also affecting fibre dispersion/distribution.

The inclusion of GP at volume fractions greater than 0.75% led to relatively lower water absorption, possibly due to the higher packing density favoured by the presence of GP and the consequent reduction in the pore diameter and interconnectivity.

### Electrical resistivity

**Fig. 8:**
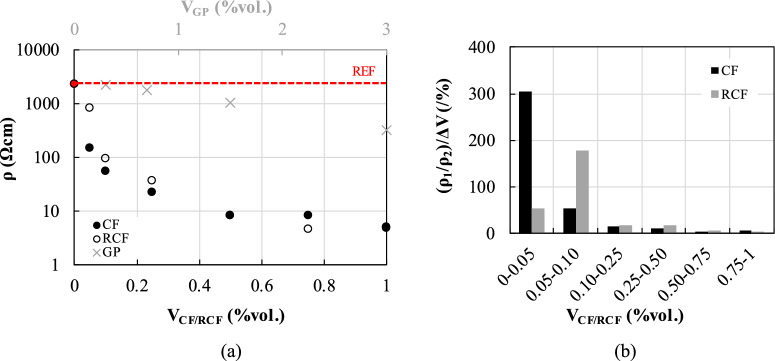
**a** Effect of inclusion content on 28-d electrical resistivity; **b** change of resistivity at each inclusion content interval, *ΔV*, versus inclusion content, only for qualitative assessment

The average 28-day electrical resistivity, *ρ*, of all doped mortars is summarized in Fig. 8a, along with that of the reference mortar, which was found to be equal to 2308.0 Ωcm. Detailed values at 7 and 28d are reported in Table 2.

The incorporation of RCF and CF resulted in a significant decrease in electrical resistivity, even at the lowest fibre content examined. For contents of 0.05%, the 28-day resistivity of RCF- and CF-doped mortars was only 36.7% and 6.6% of that of M-REF, respectively, and was further reduced by approximately two orders of magnitude for contents of 0.1%vol. (95.2 and 55.0 Ωcm for RCF ad CF, respectively). The incorporation of higher fibre contents resulted in a further reduction of the electrical resistivity, albeit not as remarkable, with minimum values attained at 0.75%vol. of RCF (4.7 Ωcm) and 1%vol. of CF (4.8 Ωcm). A remarkable reduction (up to approximately 94%) in resistivity is observed with the addition of 0.05% CF and 0.1% RCF, which is associated with the achievement of the percolation zone. This can be more clearly identified from the rate of change in resistivity observed with respect to the inclusion volume fraction ((*ρ*_1_/*ρ*_2_)/Δ*V*_CF/RCF_) (Fig. 8b). Hence, the results suggest that the percolation zone is in the range of 0.1%—0.75% for RCF-doped mortars and 0.05%—0.5% for CF-doped mortars. For higher inclusion contents, the electrical resistivity stabilises and seems to be governed by the formation of a continuous fibre network. The minimum values of CF and RCF contents for percolation identified herein are lower or comparable to those found in previous studies. This highlights the great effectiveness of RCF in developing conductive pathways in cementitious matrices, even at low concentrations.

A less significant reduction in the electrical resistivity was observed for the mortars including GP. For mortars doped with up to 0.7% vol., the 28-day resistivity was comparable to that of M-REF (−0.8% and −21.2% for 0.3% and 0.7%vol., respectively), and decreased by approximately one order of magnitude at the maximum examined content of 3%vol. (158.0 Ωcm). From these results, it is not possible to determine the percolation threshold, as higher GP contents might have resulted in a further decrease in electrical resistivity. However, as discussed in Sect. 3.1, the use of high GP contents can impact mechanical performance and higher volume fractions were deemed impractical. This is corroborated by existing evidence [12], which reported percolation at a GP content equivalent to 15% wt. of binder (much higher than the maximum 8% wt. examined in this study), albeit accompanied by a 70% reduction in compressive strength.

Taking into account that the electrical resistivity is directly related to the pore size, interconnectivity, volume fraction of pore solution and ionic concentration [46], its correlation (in terms of 28d values) with the corresponding capillary water absorption was analysed in Fig. 7b. As discussed in Sect. 3.2, high values of C indicate higher microstructural porosity and connectivity, which can promote ion mobility and concentration in the presence of water, which in turn results in a decrease in electrical resistivity. Although a clear trend between sorptivity and electrical resistivity cannot be identified, possibly due to the different tortuosity and capillary pore distribution along/across the specimens, the inherent different specimen geometry as well as the incorporation of superplasticiser, it is confirmed that in the case of CF/RCF higher values of sorptivity correspond to lower resistivity, while the opposite is observed in the case of GP-doped specimens, in which the matrix densification resulted in the reduction of the ionic conductivity.

In terms of electrical resistivity development with age, in general, 28-day old mortars were characterised by *ρ* values 20–30% higher than those at 7d. This is due to the continuous hydration of the cement particles and the resulting changes in pore structure, indicating that the conductive network in the mortar matrix relies on both ionic and electronic conduction. It was observed that at very large fibre contents (0.75%, 1%vol.), however, the 7d resistivity was only slightly lower than that at 28d, indicating the dominance of electronic rather than ionic conduction for high fibre contents (above the percolation threshold). This was not the case for mortars doped with GP, which showed a similar resistivity development with age to that of M-REF (7/28d *ρ* ratio equal to 0.75–0.80 against that of 0.83, respectively).

Decoupling ionic from electronic conductivity in self-sensing composites could also potentially provide useful insights on the quality of the microstructure, leveraging existing practice where electrical resistance measurements are used to assess the material quality and determine durability indicators. However, understanding the effect of moisture content and hydration on the electrical resistance is critical, as different environmental and curing conditions can result in different values of electrical resistance for concretes with similar microstructure [47]. The use of different types of sensors, along with the characterisation of the electrical resistance as a function of the moisture content and the development of multi-physics models focusing on electro-kinetic phenomena, should be further explored to predict material quality regardless of the electrical inclusion content and its degree of distribution within the matrix.

### Piezoresistivity

The electro-mechanical response of mortars under cyclic compressive loading was assessed through the analysis of the four metrics described in Sect. 2.2.4 (i.e. *FCR*_*max*_*, GF, L, R*^*2*^). It should be noted that *GF* was determined for specimens that exhibited a reversible change in resistivity upon strain. In general, higher values of *FCR*_*max*_ and *GF* indicate good piezoresistive performance and higher sensitivity, while *L* and* R*^*2*^ values closer to unity indicate good repeatability and high correlation between *FCR* and strain, respectively.

#### Remarks on the experimental methodology

##### Two- versus four-probe method

**Fig. 9:**
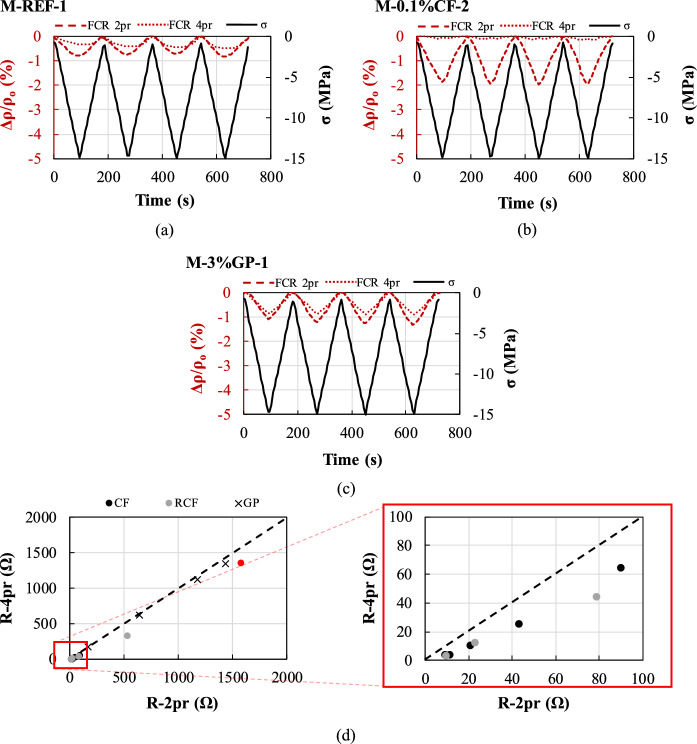
Electro-mechanical response using the two- and four-probe methods for indicative specimens with **a** no inclusions, **b** 0.1%vol. CF and **c** 3%vol. GP; **d** resistance values obtained using the two- and four-probe methods

Figure 9 presents the variation of *FCR* obtained using the two- and four-probe methods for selected representative specimens subjected to mechanical compression: a reference specimen (Fig. 9a), a specimen with 0.1% vol. CF (Fig. 9b) and a specimen with 3% vol. GP (Fig. 9c). As expected, none of the reference specimens showed substantial changes in electrical resistivity (*FCR* < 1%) as a result of the applied loading cycles. However, when using the four-probe method, minimal *FCR* was also recorded for all fibre-reinforced specimens (either CF or RCF), regardless of fibre content, with the piezoresistive performance being typically non-linear and characterised by low sensitivity (e.g. four-probe response in Fig. 9b).

While the two-probe method yielded an almost similar response for the reference specimen and the specimens doped with GP (Fig. 9a and c, respectively), the response of specimens reinforced with fibres was markedly different, with typically higher values of *FCR*_*max*_ for increasing fibre contents, and a reversible FCR-time response in accordance with the cyclic compressive stress (i.e. the electrical resistance returns to its initial state at the end of each cycle).

It could be argued that the attainment of higher *FCR*_*max*_ in the case of two-probe testing may be attributed to the electrical contact resistance (i.e. the resistance effect at the electrode/matrix interface), which can decrease with the applied strain and might result in a higher apparent resistivity under load [48]. In fact, it is confirmed in this study that the resistance measured using the two-probe method, *R-2pr*, is consistently higher than that measured using the four-probe method, *R-4pr,* (Fig. 9d). It can be also observed that the *R-2pr* values of fibre-reinforced specimens are well below the line of equality. However, the contact resistance effect remains constant across different groups of specimens and would not explain the discrepancy in terms of *FCR*_*max*_ values and piezoresistive performance between fibre- and GP-reinforced specimens, and between specimens with different fibre contents. A recent study has also provided evidence that for the same material, despite different initial resistance values were recorded using two- and four-probe configurations, similar results were yielded in terms of self-sensing behaviour [49], indicating the need for further systematic investigations to clarify the mechanisms underlying the relationship between probe arrangement and piezoresistivity.

##### Influence of electrode layout

**Fig. 10:**
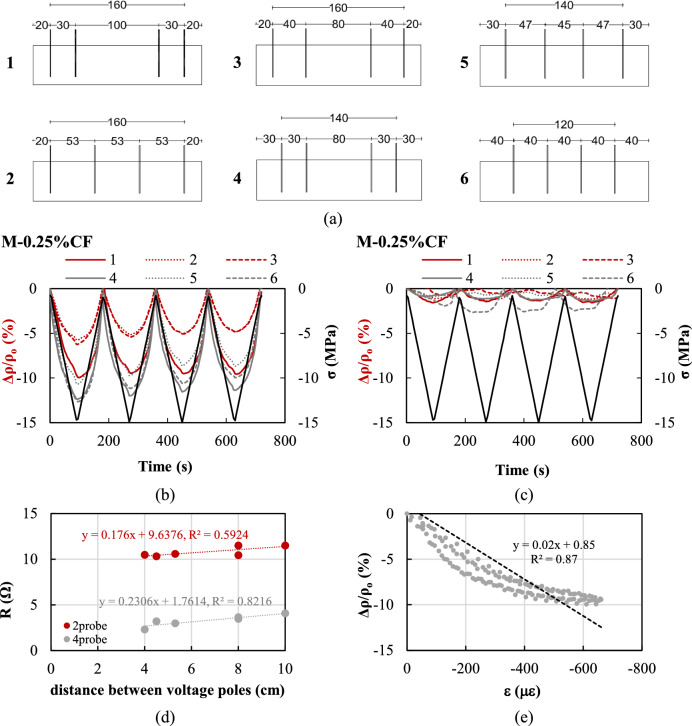
**a** Examined electrode layouts (dimensions in mm). Electro-mechanical performance of specimens with 0.25%vol. CF: **b** two-probe, **c** four-probe method; **d** effect of inter voltage pole distance on ρ_o_; **e** FCR-ε for specimen with layout #1 using the two-probe method

With the focus on the fibre-reinforced specimens, it is hypothesised that the different piezoresistive response using the two- and four-probe methods could be attributed to the electrode layout, i.e. the fact that the distance between the outer and the inner electrodes is relatively short (= 30 mm) and different local boundary conditions might have been created at the time of specimen preparation. To test this hypothesis, six additional specimens doped with 0.25%vol. of CF were cast and instrumented with different electrode layouts (Fig. 10a) to examine the effect of the distance between inner electrodes as well as the overall distance between outer electrodes. The content of CF was selected such as to achieve percolation based on the results presented in Sect. 3.3. Layouts #1, #3 and #4 comply with criteria and setups described in the literature (as discussed in Sect. 2.2.3), while #2, #5, and #6 adopt more uniform inter-electrode spacings, with different distances between the outer electrodes and the ends of the specimens. The electro-mechanical performance obtained using both two- and four-probe methods was assessed at 7d and the results are summarised in Fig. 10 and Table 3.

**Table 3:** Piezoresistive properties of specimens with different electrode layouts (absolute values)

#Layout ID	Distance between voltage poles (cm)	FCR_max_ (%)		R (Ω)		GF^*^	L^*^	R^2*^
		2pr	4pr	2pr	4pr			
1	10	10.0	1.5	11.5	4.1	162.8	0.95	0.87
2	5.3	5.7	1.2	10.6	3.0	97.4	0.86	0.86
3	8	6.2	1.1	10.4	3.5	106.2	0.77	0.84
4	8	12.4	0.7	11.5	3.7	185.0	0.92	0.85
5	4.5	10.7	2.0	10.3	3.2	165.8	0.81	0.83
6	4	12.6	2.7	10.5	2.3	201.7	0.79	0.83

^*^Calculated using the two-probe measurements

From the analysis of Fig. 10b and c, it is evident that the adoption of the four-probe method results in insufficient piezoresistive performance and low sensitivity to mechanical compressive loading, which does not seem to depend on the distance between voltage poles. In contrast, the piezoresistive response obtained using the two-probe method, is highly sensitive (*FCR*_*max*_ ranging between 5% and 13% and *GF* > 100), reversible and repeatable.

**Fig. 11:**
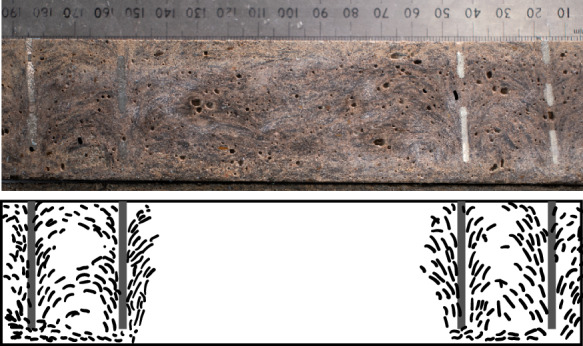
Fibre distribution between outer and inner electrodes (M-0.75%CF series)

It is therefore surmised that the adopted electrode design, layout and installation procedure resulted in a significant disturbance of the fibres distribution in the proximity of the electrodes, thus disturbing the electrical field and compromising the reliability of the four-probe method. Figure 11 presents a longitudinal cross-section of an indicative specimen with 0.75% vol. CF and schematically illustrates the spatial distribution of the fibres, clearly showing the impact that the insertion of the outer and inner electrodes during casting had on fibres alignment. This is in line with existing experimental evidence [50], which showed that a non-homogenous fibre dispersion can critically reduce the efficiency of the fibres as conductors, leading to unstable electrical and sensing behaviour. The electrodes boundary effect is considerably mitigated along the mid-portion of the specimens between inner electrodes, which is believed to result in a more uniform electric field and inter-fibre connectivity.

The implementation of inner electrodes with a reduced area such as not to obstruct the electric field (e.g. in the form of a bar), or pre-embedded electrodes at the time of casting, could have possibly alleviated this issue and resulted in more accurate measurements. Further research and microscopic analyses are needed to elucidate the effectiveness of alternative four-probe embedded configurations. Based on the results obtained in this study, a clear trend relating the effect of electrode layout to the sensitivity (*FCR*_*max*_ and *GF* -Table 3) of the tested methods cannot be readily identified. However, a dependence of resistivity on the inter-electrode spacing seems to exist, with longer distances between voltage poles resulting in an increase in resistance (Fig. 10d), albeit not significant due to the fibre content being already sufficient to provide an effective conducting network. This could be possibly attributed to the heterogeneity of the fibres within the specimens and the resulting change in the effective length, affecting the degree of inter-fibre connectivity within the composite [36].

Although the layout with equidistant inter-electrode spacing (#6) provided the highest sensitivity (*FCR*_*max*_ equal to approximately 13%), the results of this parametric study indicate that layout #1, which is the layout adopted for the remaining tests of this study, is associated with an overall superior performance and can ensure high sensitivity (*FCR*_*max*_ equal to 10%), repeatability (*L* = 0.95) and linearity between *FCR* and applied strain (*R*^*2*^ in Table 3 and Fig. 10e). The greater distance between the inner electrodes in layout #1 can also help minimise the effect of capacitance [36].

Given the aforementioned discussion, the results presented in the following section correspond to the piezoresistive performance obtained using the two-probe method. It should be noted that the results on the electrical resistivity presented in the previous section are still considered reliable, as the measurement of resistance change is not affected by the implementation of the two- or four-probe method [51].

#### Electro-mechanical behaviour of CF- and RCF-reinforced composites

**Fig. 12:**
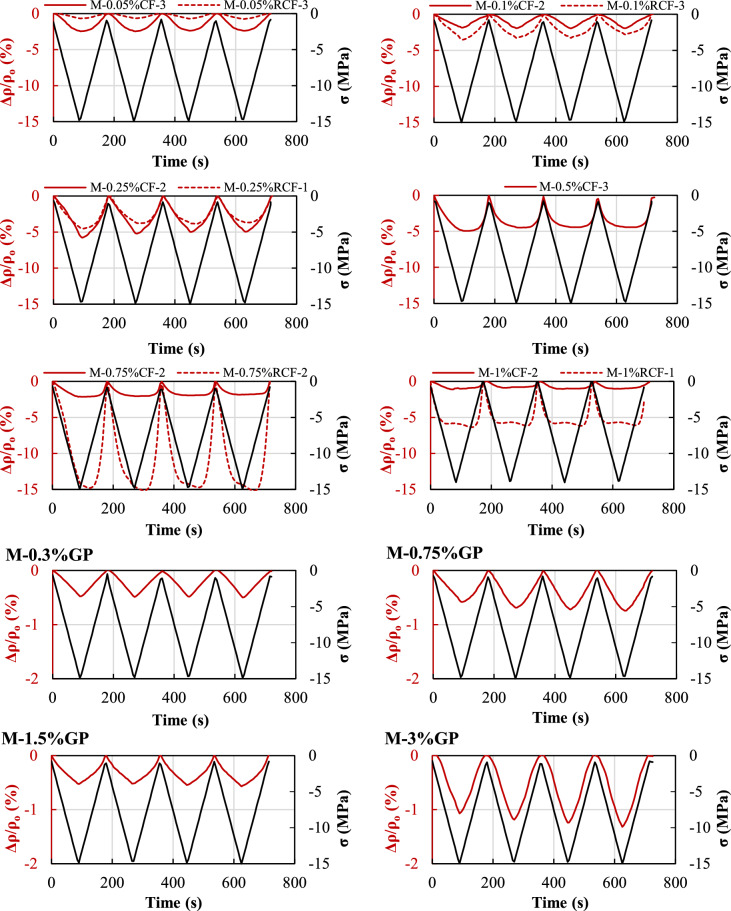
Electro-mechanical response of CF/RCF- and GP-doped specimens at all contents examined

Figure 12 shows the electro-mechanical response of CF and RCF specimens with increasing fibre content, while the associated properties are summarised in Table 4. In all cases, the resistivity decreases with the induced compressive stress and increases during unloading. As also reported in previous studies [52], with compressive loading, the proximity between the fibres increases along the direction of stress and any micro-voids or flaws are partially being closed or compressed, thus enhancing the conductivity of the composite; the opposite holds true during unloading of the specimen. The specimen without any inclusions (Fig. 9a) showed little sensitivity to the applied stress/strain (*FCR*_*max*_ equal to 1.1%), confirming the inability of conventional mortars to impart self-sensing characteristics, as recorded changes are dominated by ionic conduction and are associated with the change in capacitance and dielectric constant of the mortar matrix [53]. Specimens with 0.5%vol. of RCF showed minimal change of resistivity (*FCR*_*max*_ < 0.5%), smaller than that recorded by any other group as well as by the M-REF, which remained constant during the completion of the cycles (irreversible). This indicates presence of damage within the composite in the case of 0.5%vol. of RCF. Given that the provided content falls within the percolation zone, the lack of any piezoresistive properties is possibly attributed to problems related with significant fibre agglomeration and inadequate fibre dispersion during casting and electrode placement. Hence, these specimens are excluded from further discussion and are not taken into account in the derivation of average results.

**Table 4:** Piezoresistive properties of CF-, RCF- and GP-doped specimens (absolute values). CoV in parentheses

Specimen ID	FCR_max_ (%)	GF	L	R^2^
M-REF	1.1 (33%)	20.2 (36%)	0.84 (11%)	0.96 (2%)
M-0.05%CF	2.6 (6%)	37.8 (0%)	0.96 (2%)	0.94 (1%)
M-0.1%CF	1.7 (19%)	29.5 (14%)	0.96 (1%)	0.96 (2%)
M-0.25%CF	5.7 (2%)	106.9 (2%)	0.93 (6%)	0.85 (7%)
M-0.5%CF	3.8 (41%)	–	0.90 (4%)	0.67 (0%)
M-0.75%CF	2.6 (22%)	–	0.88 (4%)	0.61 (1%)
M-1%CF	1.2 (1%)	–	0.83 (9%)	0.52 (49%)
M-0.05%RCF	0.7 (10%)	11.4 (6%)	0.94 (3%)	0.91 (3%)
M-0.1%RCF	3.6 (4%)	58.8 (10%)	0.87 (2%)	0.88 (4%)
M-0.25%RCF	5.3 (21%)	90.2 (17%)	0.82 (6%)	0.80 (3%)
M-0.5%RCF^*^	0.3 (5%)	–	–	0.25 (6%)
M-0.75%RCF	17.8 (16%)	–	0.95 (4%)	0.59 (16%)
M-1%RCF	5.2 (33%)	–	0.96 (2%)	0.50 (13%)
M-0.3%GP	0.5 (10%)	8.2 (12%)	0.90 (12%)	0.98 (1%)
M-0.75%GP	0.8 (11%)	12.2 (8%)	0.87 (2%)	0.93 (3%)
M-1.5%GP	0.7 (19%)	8.0 (15%)	0.85 (15%)	0.91 (9%)
M-3%GP	1.2 (8%)	12.7 (13%)	0.89 (0%)	0.95 (5%)

^*^Excluded from discussion

The incorporation of increased fibre contents had a significant effect on the sensitivity, reversibility and linearity of the piezoresistive behaviour. The electro-mechanical performance of composites doped with contents ≤ 0.25%vol. CF/RCF was reversible and characterised by nearly stable values of resistivity under maximum and minimum stress between subsequent cycles (*L* > 0.86), indicating good repeatability, and linear change of *FCR* with induced strain (*R*^*2*^ > 0.80), confirming that no internal damage occurred under cyclic compressive loading. Specimens doped with CF exhibited sufficient sensitivity even at the lowest content examined (0.05%vol.), while the minimum content required to provide similar piezoresistivity in composites doped with RCF was equal to 0.10%. This is in agreement with the results obtained from the electrical resistivity characterisation tests (Fig. 8), confirming that percolation has been reached. Despite their substantially lower electrical resistivity compared to that of the M-REF, composites with contents < 0.1% vol. RCF resulted in composites with poor sensing behaviour.

The highest piezoresistivity was achieved by composites doped with 0.25% vol. CF and 0.75% vol. RCF. For the former, *FCR*_*max*_ was equal to 5.7% and the associated *GF* equal to 107. Equally sensitive piezoresistive performance was obtained by the RCF counterparts, with associated *FCR*_*max*_ and *GF* values equal to 5.3% and 90, respectively. Composites with sensitivity of such magnitude are sufficiently sensitive to the change in the applied stress/strain and can be used as piezoresistive sensors [5], thus highlighting the potential of recycled carbon fibres in substituting carbon fibres in smart applications. The values of *FCR*_*max*_ obtained for 0.25%vol. are slightly higher than those obtained in [23] (2.5 and 3.5 at 0.20%vol. CF and RCF, respectively), while the associated GF values are considerably lower than those in [23] (163–651), where the non-linearity of the dependence of the resistivity on strain is considered for the derivation of *GF*.

It should be noted that the values of *FCR*_*max*_ and *GF* obtained for 0.25%vol. CF are lower than those reported in the previous section (Fig. 10b, Table 3), due to the effect of curing time on the piezoresistive properties and the ongoing hydration: 7-day old composites have a larger amount of free water, lower stiffness and have experienced lower autogenous shrinkage than the 28-day old counterparts. The more sensitive self-sensing behaviour of early-age composites has also been highlighted in previous studies [54].

RCF-doped composites with 0.75%vol. exhibited the highest *FCR*_*max*_ (16%) among all examined composites and an overall reversible behaviour. However, *FCR* appeared to be less sensitive to the maximum applied strain and a loss of linearity within the applied strain range was recorded, resulting in a state of “balance” in the proximity to the maximum applied strain. This state was also apparent and more pronounced at both CF and RCF fibre contents higher than the one resulting in the highest *FCR*_*max*_ (R^2^ values ranging between 0.5–0.7). This is possibly due to the germination of microcracks, which resulted in a destruction of existing conductive paths and reconstruction of new ones, while tunnelling conduction was still guaranteed. Composites with contents CF > 0.25%vol. and RCF > 0.75%vol. presented lower sensitivity indicating that these values of fibre content demarcate the end of the percolation zone, and the formation of a stable conductive network (contacting conduction). Despite the similar mechanical and electrical properties of CF/RCF composites, RCF were proven effective in imparting self-sensing ability over a higher range of fibre contents, indicating their superiority over CF in diverse applications (e.g. de-icing). This may be possibly attributable to a better dispersion/distribution of RCF within the matrix and the development of more effective microstructural electrokinetic mechanisms. Additional microstructural analyses are needed to further clarify these aspects, especially concerning the inherent morphology of RCF from different origin and recycling processes.

**Fig. 13:**
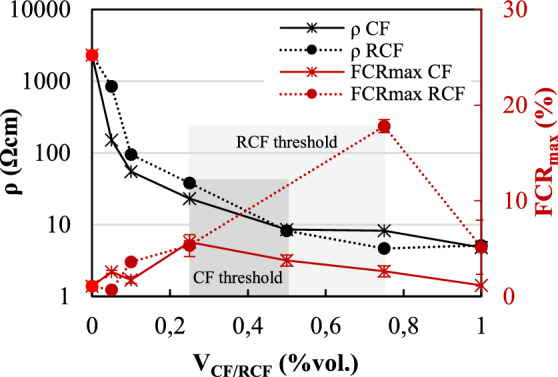
Effect of CF/RCF content on the resistivity: identification of the percolation transition zone

Based on the discussion above, the use of 0.25%vol. of CF/RCF provides the desirable synergy between structural performance, cost and self-sensing properties. It also suggested that the percolation threshold for each inclusion can be identified within the zone demarcated by the inclusion contents that ensure a combination of low *ρ* (e.g. two orders of magnitude lower than that of the reference sample) and maximisation of *FCR*_*max*_. An illustration for the determination of CF and RCF percolation threshold is presented in Fig. 13, according to which it is found to be between 0.25 and 0.5%vol. and 0.25–0.75%vol., respectively.

#### Electro-mechanical behaviour of composites doped with GP

The electromechanical performance of specimens doped with GP under compressive cyclic loading is summarized in Fig. 12 and Table 4. Although all specimens were characterised by a cyclic change of electrical resistivity that was highly linear with the applied compressive stress (*R*^2^ > 0.91) and highly repeatable between consecutive cycles (*L* values ranging between 0.85 and 0.90), a lower sensitivity than that attained by the M-REF was recorded. Only specimens doped with 3%vol. achieved a slightly better performance, though characterised by poor variability and *GF* values (between 8 and 13). While the underlying sensing mechanism is similar to that described for the case of fibre inclusions, i.e. the distance between graphite particles reduces upon the application of compressive stress resulting in a reduction of electrical resistivity, piezoresistive properties and a self-sensing functionality could not be achieved for GP-doped specimens. This is not surprising, as all composites were in the pre-percolation zone (Sect. 3.3), where ionic conduction is more prevalent and the distance between graphite particles is not small enough to guarantee tunnelling conduction. The values of *FCR*_*max*_ and *GF* at the best conditions (i.e. at 3%vol.) are lower than those reported in the literature for expanded graphite at lower contents. For instance, in [14] a *GF* = 68 was achieved at GP content of 5% wt. cement after performing tests on dry composites; these are typically characterised by higher piezoresistive performance when compared to fully saturated specimens, due to the elimination of the water content and the resulting decrease of the contact resistance between fillers [55].

## Conclusions

This paper presents the results of a systematic study on the performance of cementitious mortars doped with virgin/recycled CF and GP. Key underlying microstructural mechanisms as well as challenges associated with existing testing protocols were identified and discussed. The following conclusions can be drawn:

- The effect of virgin and recycled CF on the mechanical and transport properties was similar. An increase in fibre content resulted in almost linear increase in flexural strength, with both types of fibres able to effectively control crack development.
- Virgin and recycled CF were highly effective in reducing the electrical resistivity of mortar composites. At contents as low as 0.1%vol., the resistivity of RCF and CF is 95.2 and 55.0 Ωcm, only 4% and 2.4% of that of the reference specimens, respectively.
- The percolation threshold can be identified within the limits ensuring low resistivity and maximum sensitivity. This was found to lie between 0.25–0.5%vol. and 0.25–0.75%vol., for CF and RCF, respectively.
- The use of 0.25%vol. of CF/RCF was found to provide the desirable synergy between structural performance, cost and self-sensing properties. Resulting mortars have 50–60% higher flexural strength, and good piezoresistive properties (*GF* in the range of 90–110, *FCR*_*max*_ up to 6%).
- The addition of GP as sand replacement resulted in an almost proportional decrease in compressive strength (up to 33% for 3%vol.) and composites in the pre-percolation zone with poor self-sensing behaviour.
- Non-uniform fibre dispersion can critically affect mechanical strength and the efficiency of the fibres as conductors. Optimisation of fibre dispersion, through the use of compatible dispersants at different stages of mixing or preparation, or through the use of appropriate sizing during manufacture, is imperative to unlock the full potential of virgin and recycled carbon fibres in cementitious composites.
- The reliability of piezoresistive measurements obtained with the four-probe method may be compromised by uneven fibre dispersion between outer and inner electrodes at their proximity, which can disturb the electric field and reduce inter-fibre connectivity. Existing testing protocols should be revisited, while alternative configurations should be examined and proposed as a function of the inclusion type.

The findings of this study demonstrate that RCF can serve as an effective alternative to virgin CF in smart structural applications. Indeed, it was shown that the use of RCF does not hinder the electrical and piezoresistive performance, while still offering mechanical properties that are on par with those of their non-recycled counterparts. Future research is essential to verify and broaden the applicability of the findings to mixes with RCF obtained from different sources or processes. This includes detailed microstructural analyses to assess fibre dispersion and its effect on self-sensing performance, as well as the assessment of the self-sensing ability provided by the fibres under different loading conditions and beyond the elastic limits, on both un-damaged and damaged structural elements, with a view to detect and identify damage in more realistic scenarios. The synergistic effect of sustainable conductive inclusions and supplementary cementitious materials on the combined electro-mechanical properties should be also further explored to fine-tune the mix design for optimal sensing. Finally, the durability of these alternative, sustainable “smart” composites should be assessed to facilitate their practical implementation in real-world applications.

The reliability of the piezoresistive measurements obtained with the two- and the four-probe methods bears a challenge in field applications as access to structural elements for sensor installation is not always feasible, whereas typically employed four-sensor arrays only provide surface measurements and are susceptible to local defects. Electrical Resistance Tomography, enabled through the use of a two- or three-dimensional arrays of electrodes (attached or embedded) is a promising emerging technique that allows for spatial resistivity measurements and distributed sensing, thus unlocking the potential of alternative inclusions in structural health monitoring and quality control in traditional construction as well as in additive manufacturing.
